# Subspecies-specific metabolic gene clusters shape polyphenol dynamics for modular metabolic breeding in sugarcane

**DOI:** 10.1016/j.xplc.2026.102042

**Published:** 2026-08-06

**Authors:** Dongdong Wang, Ding Fan, Jisen Zhang

**Affiliations:** 1 Guangxi Sugarcane Bio-Breeding Laboratory, State Key Lab for Conservation and Utilization of Subtropical Agro-Biological Resources, College of Agriculture, Guangxi University, Nanning, China

## Main text

Modern sugarcane cultivars are highly polyploid interspecific hybrids derived predominantly from *Saccharum officinarum* and *Saccharum spontaneum*. These divergent subgenomic contributions support high sugar yields and environmental resilience; however, they also complicate efforts to combine productivity with durable stress tolerance (Wang et al., 2023; Zhang et al., 2025). The multiscale graph pan-genome and dosage-aware genome-wide association study (DosageGWAS) framework recently developed for sugarcane now permits the analysis of subgenome-resolved variants and the quantitative estimation of allele dosage (Huang et al., 2026). This framework builds on rice pan-genome studies that revealed extensive sequence, presence/absence, and structural variation across cultivated and wild germplasm and established a super pan-genomic landscape (Zhao et al., 2018; Shang et al., 2022), while extending graph-based representation to mixed-ploidy sugarcane. Together, these resources create an opportunity to examine whether polyphenol biosynthetic genes and their regulators form subspecies-biased metabolic modules that influence the growth–defense balance.

Polyphenols participate in redox regulation, stress adaptation, and developmental signaling. In sugarcane, MYB- and WRKY-family regulators have been characterized, but the physical organization, ancestry, and dosage dependence of the corresponding pathway genes remain incompletely resolved (Li et al., 2020; Yuan et al., 2021). We therefore propose a testable framework in which candidate subspecies-specific metabolic gene clusters (MGCs) coordinate polyphenol dynamics through genomic organization, transcriptional regulation, biochemical feedback, and allele-dosage effects. We also discuss how these layers could inform modular metabolic breeding. Throughout, we distinguish established observations from hypotheses that require direct validation in sugarcane. Supplemental Table S1 defines the principal terms used in this framework.

## Genomic architecture and transcriptional regulatory networks of subspecies-specific MGCs

**Figure 1:**
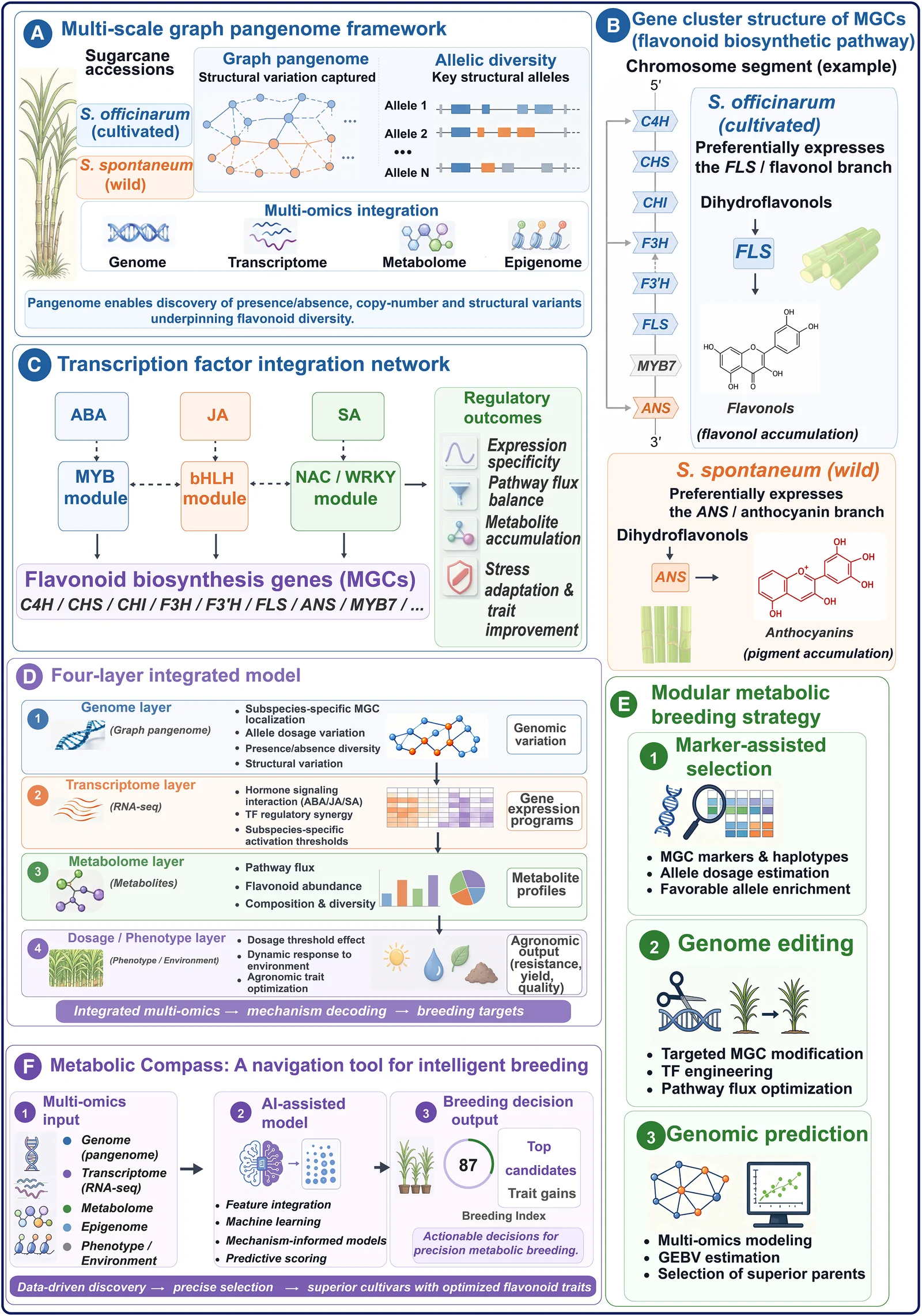
An integrated framework for testing subspecies-specific flavonoid metabolic gene clusters (MGCs) and guiding modular metabolic breeding in sugarcane. **(A)** A multiscale graph pan-genome mapping alleles and structural variants to ancestral *Saccharum* backgrounds and integrating genomic variation with transcriptomic, metabolomic, and epigenomic information. **(B)** Candidate pathway modules comprising an *S. officinarum*-biased FLS/flavonol branch and an *S. spontaneum*-biased ANS/anthocyanin branch. These branch assignments are working hypotheses rather than validated MGC boundaries. **(C)** ABA, jasmonic acid, and salicylic acid signaling may converge on MYB, basic helix-loop-helix, NAC, and WRKY regulators to influence pathway activity, metabolite accumulation, and stress responses. **(D)** A four-layer model integrating genomic architecture and dosage, transcriptional regulation, metabolic output, and agronomic phenotype. **(E)** Proposed breeding workflow combining module identification, targeted editing or *cis*-regulatory engineering, and dosage-aware genomic prediction. Recombination, linkage, and interactions among ancestral alleles must be considered in module design. **(F)** The “metabolic compass” integrates basal metabolite abundance, stress-induced response amplitude, allele dosage, environment, and agronomic performance to inform population- and environment-specific predictions. This conceptual decision framework does not prescribe a universal ancestral dosage ratio.

The sugarcane graph pan-genome projects nine genomes from four *Saccharum* species onto 10 ancestral chromosome communities, enabling alleles and structural variants to be assigned to their likely ancestral backgrounds (Huang et al., 2026). This coordinate system enables testing whether flavonoid pathway genes are physically clustered, co-inherited, or coordinately regulated in a subspecies-dependent manner. Figures 1A and 1C present two working modules: an *S. officinarum*-biased module associated with the FLS/flavonol branch and an *S. spontaneum*-biased module associated with the ANS/anthocyanin branch. These assignments represent working hypotheses rather than established MGC boundaries.

Validation requires more than colocalization. Candidate modules should be evaluated using haplotype-resolved structural variation, linkage and recombination patterns, chromatin accessibility, transcription-factor occupancy, co-expression, and metabolite quantitative-trait loci. Their activity should also be tested across tissues, developmental stages, and stress conditions. ABA, jasmonic acid, and salicylic acid signaling may converge on MYB, basic helix-loop-helix, NAC, and WRKY regulators, thereby altering pathway flux and metabolite accumulation. Sugarcane MYB and WRKY families provide plausible regulatory candidates (Li et al., 2020; Yuan et al., 2021); however, direct connections between specific hormone-responsive factors, ancestral haplotypes, and metabolic outputs remain to be demonstrated. A rigorous MGC designation should therefore require evidence of physical proximity or conserved linkage, as well as coordinated regulation and a shared metabolic function.

## Putative polyphenol–hormone–ion regulatory mechanisms

In addition to serving as pathway end products, polyphenols may modulate developmental and stress signaling. In *Arabidopsis*, flavonols stabilize PIN auxin-efflux complexes and influence polar auxin transport, providing a mechanistic precedent for feedback from specialized metabolism to growth regulation (Teale et al., 2020). Stress-induced ABA signaling may likewise increase flavonol production through SnRK2-, ABF/AREB-, and MYB-associated transcriptional programs, and accumulated flavonols can in turn alter cellular redox status. However, a direct ABA–flavonol feedback loop has not been established in sugarcane.

Guard-cell ion transport is an additional plausible interface between polyphenol accumulation and stress responses (Kashtoh and Baek, 2021). Ca^2+^–calmodulin signaling is an established component of plant immune activation and may provide a second route linking metabolic state to stomatal or immune responses (Sun et al., 2022), but direct regulation by sugarcane polyphenols remains unproven. Supplemental Figure 1 therefore presents these interactions as testable mechanisms inferred largely from model plants. Priority experiments include ligand–protein interaction assays, channel electrophysiology, phosphoproteomics, targeted gene perturbation, and metabolite add-back experiments. These experiments will help distinguish direct biochemical regulation from indirect effects arising from altered redox state, hormone abundance, or transcriptional reprogramming.

## Quantitative modeling of polyploid dosage effects

In a highly polyploid genome, the presence or absence of an allele is less informative than its copy number, ancestry, and regulatory context. By estimating continuous allele dosage, DosageGWAS increased the proportion of heritability explained for representative sugarcane traits, demonstrating the analytical power of dosage-aware models (Huang et al., 2026). However, gene dosage may not translate linearly into transcript or metabolite abundance. Polyploid systems can display proportional expression, dosage compensation, homoeolog bias, and pathway-level constraints (Bekaert et al., 2011; He et al., 2022).

For candidate MGCs, the relationships between dosage and expression, metabolite abundance, and phenotype should therefore be modeled using nonlinear or piecewise functions rather than by assuming a universal threshold. Models should incorporate dosage uncertainty, ancestral origin, population structure, environment, dosage-by-environment interactions, and epistasis among homoeologs. Basal polyphenol abundance and the amplitude of stress-induced responses may serve as complementary quantitative traits: high constitutive abundance could impose a metabolic cost, whereas a rapid inducible pulse may preserve defense with lower basal expenditure. The proposed 60% *S. spontaneum* dosage threshold and 40:60 ancestral ratio shown in Supplemental Figure 2 are illustrative, hypothesis-generating values rather than validated optima. Locus-specific thresholds must be estimated empirically and replicated across genetic backgrounds and environments.

## An integrated model and modular metabolic breeding strategy

The proposed framework links four analytical layers (Figure 1D). The genome layer defines candidate MGC locations, ancestry, haplotype structure, and dosage. The transcriptional layer measures hormone-responsive regulatory wiring and activation thresholds. The metabolic layer quantifies pathway flux, metabolite composition, basal abundance, and inducible responses. The phenotype layer evaluates yield, sugar accumulation, quality, and stress performance. Integrating these layers can transform a descriptive pathway model into a set of testable, potentially selectable modules.

A modular metabolic breeding workflow would proceed in three stages (Figure 1E). First, graph pan-genome, transcriptomic, and metabolomic data would be integrated to identify candidate modules and prioritize alleles with reproducible effects. Second, genome editing or *cis*-regulatory engineering would be used to modify specific nodes while preserving the remainder of the pathway (Li et al., 2024). Regulatory-element editing has been used to decouple yield components in rice, and targeted editing of metabolic pathways has altered gossypol composition in cotton, demonstrating the feasibility of trait-specific metabolic redesign (Song et al., 2022; Lin et al., 2023). In sugarcane, editing strategies must account for allele dosage, homoeolog redundancy, off-target effects, and the possibility that linked genes within an MGC may be inherited as a unit. Recombination frequency and epistatic interactions among ancestral alleles should therefore also be considered in module design.

Third, dosage-aware genomic prediction could evaluate combinations that are impractical to test exhaustively in the field. We term the resulting decision framework a “metabolic compass” (Figure 1F and Supplemental Figure 3): a model that combines basal metabolic levels, stress-induced response amplitude, allele dosage, environment, and agronomic performance. It should not prescribe a universal ancestral ratio. Instead, it should generate uncertainty-calibrated predictions for specific populations and target environments. CRISPR and other genome-modification tools can then be used to test high-priority predictions rather than directly implementing unvalidated dosage targets. Prospective validation should compare edited or selected lines across environments and measure both agronomic benefits and potential metabolic costs. An illustrative implementation roadmap and AI-assisted prediction workflow are presented in Supplemental Figure 3.

## Concluding remarks

The sugarcane graph pan-genome and DosageGWAS framework enable polyphenol regulation to be investigated at subgenome and copy-number resolution. We propose that candidate subspecies-specific MGCs may link ancestral genome structure to transcriptional regulation, metabolic feedback, and quantitative dosage effects. This model offers a coherent route from association to mechanism, but its central elements—including MGC boundaries, branch-specific ancestry, hormone–ion feedback, and dosage thresholds—remain hypothetical and require experimental validation.

Modular metabolic breeding should therefore follow an iterative design–test–learn strategy. Its success will depend on accurate dosage estimation, causal validation, explicit modeling of recombination and allele interactions, and field testing across environments. If these requirements are met, the framework could help identify polyphenol configurations that preserve inducible defense while limiting unnecessary constitutive metabolic costs, thereby supporting precision improvement in complex polyploid crops.

## Acknowledgments

We thank the editor and three reviewers for their constructive evaluation of this commentary. We also thank Muqing Zhang and colleagues for discussions that informed the polyphenol–hormone–ion framework, the Sugarcane Breeding Innovation Team at Guangxi University for insights into polyploid genetics and dosage effects, and Jishuang Chen for comments on MGCs.

Funding: this work was supported by the National Natural Science Foundation of China (grant no. U24A20387 to J.Z.), the Guangxi Science and Technology Major Program (grant no. AB24153006 and AA24206023 to J.Z.), and the National Key Research and Development Program (grant no. 2024YFF1000800).

## Author contributions

J.Z., conceptualization, supervision, funding acquisition, and writing – review and editing; D.W., investigation, data curation, formal analysis, and writing – original draft; D.F., methodology, investigation, and writing – review and editing. All authors have read and approved the manuscript.

## Supplemental information

Document S1. Figures S1–S3, Table S1, Notes S1–S3, and supplemental information

Document S2. Article plus supplemental information
