# Supplementary material for: Subspecies-specific metabolic gene clusters shape polyphenol dynamics for modular metabolic breeding in sugarcane
Source: Plant Commun. 2026 Aug 6;7(9):102042. doi: 10.1016/j.xplc.2026.102042 (PMC13589566; doi:10.1016/j.xplc.2026.102042)

Plant Communications, Volume 7

Supplemental information

Subspecies-specific metabolic gene clusters shape polyphenol dynamics for modular metabolic breeding in sugarcane

Dongdong Wang, Ding Fan, and Jisen Zhang

Supplemental Information

Subspecies-specific metabolic gene clusters orchestrate polyphenol dynamics for modular metabolic breeding in sugarcane

Dongdong Wang[1], Ding Fan[1], Jisen Zhang[1]*

[1] Guangxi Sugarcane Bio-Breeding Laboratory, State Key Lab for Conservation and Utilization of Subtropical Agro-Biological Resources, College of Agriculture, Guangxi University, Nanning, China

*Correspondence: Jisen Zhang (zjisen@gxu.edu.cn)

Supplemental Note S1. Evidence and validation requirements for putative polyphenol–hormone–ion interactions

Figure S1 separates evidence established in model plants from mechanisms proposed for sugarcane. Flavonol-dependent stabilization of PIN auxin-efflux complexes provides direct evidence that a specialized metabolite can alter hormone transport (Teale et al., 2020). This observation supports, but does not demonstrate, a comparable flavonol–auxin module in sugarcane. Validation should combine sugarcane PIN homolog identification, subcellular localization, phosphosite analysis, ligand-binding assays and auxin-transport measurements after genetic or chemical manipulation of flavonol abundance.

ABA-associated flavonol accumulation is presented as a regulatory hypothesis. A defensible test should distinguish transcriptional induction of biosynthesis from direct modulation of ABA receptors or signaling proteins. Time-resolved ABA, ROS and flavonol measurements, together with perturbation of PYR/PYL–PP2C–SnRK2 and MYB/ABF components, would establish directionality. Rescue experiments should determine whether altered flavonol abundance restores the relevant phenotype independently of broader stress-induced transcriptional changes.

Guard-cell ion transport is a plausible interface because K^+ and other channels regulate

stomatal responses (Kashtoh and Baek, 2021). Ca^{2+}–calmodulin signaling is required for important plant immune outputs and provides an additional testable route linking metabolic state with stress signaling (Sun et al., 2022), but direct control by sugarcane polyphenols remains unproven. Electrophysiology, purified-protein binding, dose–response analysis and genetic epistasis are required before these interactions can be described as causal sugarcane mechanisms.

Supplemental Note S2. Assumptions for dosage-aware analysis of candidate MGCs

DosageGWAS provides a quantitative basis for associating allele copy number with complex traits in mixed-ploidy sugarcane (Huang et al., 2026). For candidate MGCs, however, dosage should be propagated as an estimate with uncertainty rather than forced into an error-free integer. Models should jointly consider ancestry, local haplotype structure, population structure, environmental covariates and relatedness.

The expected relationship among dosage, expression and metabolite abundance may be linear, saturating, threshold-like or compensated. Evidence from polyploid metabolic networks and wheat homoeolog expression shows that dosage sensitivity is locus- and pathway-dependent (Bekaert et al., 2011; He et al., 2022). Accordingly, linear, spline and piecewise models should be compared, with out-of-sample validation and uncertainty intervals. Dosage-by-environment and dosage-by-dosage interactions should be tested when sample size permits.

Figure S2 uses basal abundance and stress-induced pulse amplitude as conceptual phenotypes. The displayed 60% *S. spontaneum* value is an illustrative reference point, not a threshold established by Huang et al. (2026) or by direct sugarcane CHS experiments. Likewise, a 40:60 *S. officinarum*:*S. spontaneum* ratio must not be interpreted as a universal breeding optimum. Any threshold or optimum must be estimated at a defined locus, in a defined population and environment, and replicated independently.

Supplemental Note S3. Implementation of modular metabolic breeding and the metabolic compass

The proposed workflow has three iterative stages. Module identification integrates graph-pangenome haplotypes, structural variants, expression, chromatin features, metabolite profiles and phenotypes, building on chromosome-resolved Saccharum genomic resources (Wang et al., 2023; Huang et al., 2026). Candidate modules should be prioritized only when ancestry and dosage effects are reproducible and when physical linkage or coordinated regulation supports an MGC interpretation.

Module design uses genome editing, cis-regulatory engineering or allele selection to perturb a defined node (Li et al., 2024). Examples in rice and cotton show that regulatory and metabolic traits can be redesigned without altering all pathway functions (Song et al., 2022; Lin et al., 2023). Sugarcane applications must additionally address high ploidy, homoeolog redundancy, mosaic editing and linkage among neighboring genes. Each design therefore requires molecular characterization, dosage verification and multi-environment field validation.

Module assembly combines dosage-aware prediction with multi-environment phenotyping. The metabolic compass should output predicted benefit, metabolic cost and uncertainty rather than a single fixed ratio. Training and validation datasets must be separated, and model performance should be evaluated across genetic backgrounds and target environments. The timelines shown in Figure S3 are illustrative planning scenarios rather than demonstrated reductions in breeding duration.

Supplemental Table S1. Glossary of key terms for modular metabolic breeding

Term	Definition
Metabolic gene cluster (MGC)	A physically linked or conserved set of genes that contributes to a shared metabolic function. In this Commentary, the proposed sugarcane MGCs remain candidate modules pending structural and functional validation.

Term	Definition
DosageGWAS	A dosage-aware association framework that estimates allele dosage in polyploid genomes and tests its relationship with quantitative traits.
Basal metabolic level	Metabolic abundance under a defined non-stressed condition. Its interpretation requires standardized tissue, developmental stage and environment.
Response pulse amplitude	The stress-induced change relative to the basal level, quantified over a defined time course.
Metabolic compass	A conceptual prediction framework integrating metabolic state, allele dosage, environment and agronomic performance with calibrated uncertainty.
Yield–defense balance point	A population-, locus- and environment-specific compromise between productivity and defense. It is not assumed to correspond to a universal ancestral dosage ratio.

Supplementary Figure legends

Supplementary Figure S1. Putative polyphenol-mediated regulation of hormone and ion signaling.

A. Model-plant evidence indicates that flavonols can stabilize PIN auxin-efflux complexes and alter polar auxin transport. B. ABA signaling may induce flavonol biosynthesis, whereas flavonol-dependent redox changes may feed back on stress responses. C. Guard-cell K^+ channels and Ca^{2+}–calmodulin signaling are proposed as additional interfaces. The pathways in B and C remain to be validated directly in sugarcane.

Supplementary Figure S2. Conceptual modeling of polyploid dosage effects.

A. DosageGWAS estimates continuous allele dosage and tests dosage–trait associations. B. A nonlinear dosage–metabolite relationship is shown as an illustrative model; the plotted 60% *S. spontaneum* value is not a validated CHS threshold. C. Basal metabolic level and stress-induced pulse amplitude are proposed as complementary quantitative traits. The direction and magnitude of these relationships must be estimated empirically for each locus and environment.

Supplementary Figure S3. Implementation roadmap and AI-assisted metabolic compass for modular metabolic breeding in sugarcane.

A. Comparison of a conventional breeding workflow with a proposed modular workflow comprising module identification, module design and module assembly. The displayed durations are illustrative planning scenarios, not demonstrated reductions in breeding time. B. The metabolic compass integrates basal metabolic level, response pulse amplitude, allele dosage, environment and field performance in an uncertainty-calibrated prediction model.

Supplemental References

Bekaert, M., Edger, P.P., Pires, J.C., and Conant, G.C. (2011). Two-phase resolution of polyploidy in the *Arabidopsis* metabolic network gives rise to relative and absolute dosage constraints. Plant Cell 23, 1719–1728. https://doi.org/10.1105/tpc.110.081281.

He, F., Wang, W., Rutter, W.B., Jordan, K.W., Ren, J., Taagen, E., et al. (2022). Genomic variants affecting homoeologous gene expression dosage contribute to agronomic trait variation in allopolyploid wheat. Nature Communications 13, 826. https://doi.org/10.1038/s41467-022-28453-y.

Huang, Y., Zhang, Y., Zhang, Q., Zhuang, G., Li, C., Wang, B., et al. (2026). Multiscale pangenome graphs empower the genomic dissection of mixed-ploidy sugarcane species. Science 391, eadx1616. https://doi.org/10.1126/science.adx1616.

Kashtoh, H., and Baek, K.-H. (2021). Structural and functional insights into the role of guard cell ion channels in abiotic stress-induced stomatal closure. Plants 10, 2774. https://doi.org/10.3390/plants10122774.

Li, B., Sun, C., Li, J., and Gao, C. (2024). Targeted genome-modification tools and their advanced applications in crop breeding. Nature Reviews Genetics 25, 603–622. https://doi.org/10.1038/s41576-024-00720-2.

Li, Z., Hua, X., Zhong, W., Yuan, Y., Wang, Y., Wang, Z., et al. (2020). Genome-wide identification and expression profile analysis of WRKY family genes in the autopolyploid *Saccharum spontaneum*. Plant and Cell Physiology 61, 616–630. https://doi.org/10.1093/pcp/pcz227.

Lin, J.-L., Fang, X., Li, J.-X., Chen, Z.-W., Wu, W.-K., Guo, X.-X., et al. (2023). Dirigent gene editing of gossypol enantiomers for toxicity-depleted cotton seeds. Nature Plants 9, 605–615. https://doi.org/10.1038/s41477-023-01376-2.

Shang, L., Li, X., He, H., Yuan, Q., Song, Y., Wei, Z., et al. (2022). A super pan-genomic landscape of rice. Cell Research 32, 878–896. https://doi.org/10.1038/s41422-022-00685-z.

Song, X., Meng, X., Guo, H., Cheng, Q., Jing, Y., Chen, M., et al. (2022). Targeting a gene regulatory element enhances rice grain yield by decoupling panicle number and size. Nature Biotechnology 40, 1403–1411. https://doi.org/10.1038/s41587-022-01281-7.

Sun, L., Qin, J., Wu, X., Zhang, J., and Zhang, J. (2022). TOUCH 3 and CALMODULIN 1/4/6 cooperate with calcium-dependent protein kinases to trigger calcium-dependent activation of CAM-BINDING PROTEIN 60-LIKE G and regulate fungal resistance in plants. Plant Cell 34, 4088–4104. https://doi.org/10.1093/plcell/koac209.

Teale, W.D., Pasternak, T., Dal Bosco, C., Dovzhenko, A., Kratzat, K., Bildl, W., et al. (2020). Flavonol-mediated stabilization of PIN efflux complexes regulates polar auxin transport. EMBO Journal 40, e104416. https://doi.org/10.15252/embj.2020104416.

Wang, T., Wang, B., Hua, X., Tang, H., Zhang, Z., Gao, R., et al. (2023). A complete gap-free diploid genome in *Saccharum* complex and the genomic footprints of evolution in the highly polyploid *Saccharum* genus. Nature Plants 9, 554–571. https://doi.org/10.1038/s41477-023-01378-

0.

Yuan, Y., Yang, X., Feng, M., Ding, H., Khan, M.T., Zhang, J., et al. (2021). Genome-wide analysis of R2R3-MYB transcription factors family in the autopolyploid *Saccharum spontaneum*: an exploration of dominance expression and stress response. BMC Genomics 22, 622. https://doi.org/10.1186/s12864-021-07689-w.

Zhang, J., Qi, Y., Hua, X., Wang, Y., Wang, B., Qi, Y., et al. (2025). The highly allo-autopolyploid modern sugarcane genome and very recent allopolyploidization in *Saccharum*. Nature Genetics 57, 242–253. https://doi.org/10.1038/s41588-024-02033-w.

Zhao, Q., Feng, Q., Lu, H., Li, Y., Wang, A., Tian, Q., et al. (2018). Pan-genome analysis highlights the extent of genomic variation in cultivated and wild rice. Nature Genetics 50, 278–284. https://doi.org/10.1038/s41588-018-0041-z.

Supplementary Figure S1.

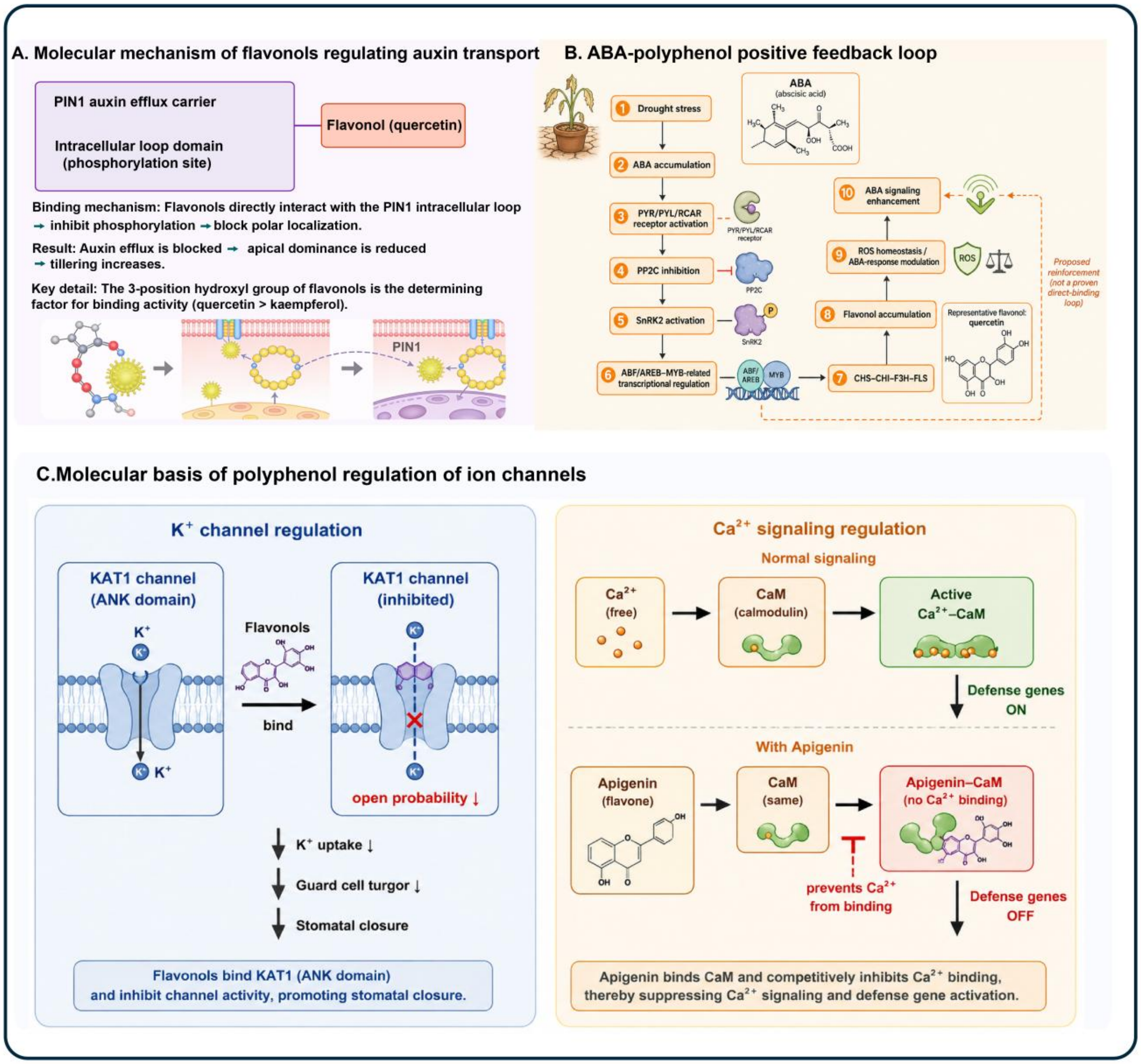

Supplementary Figure S2.

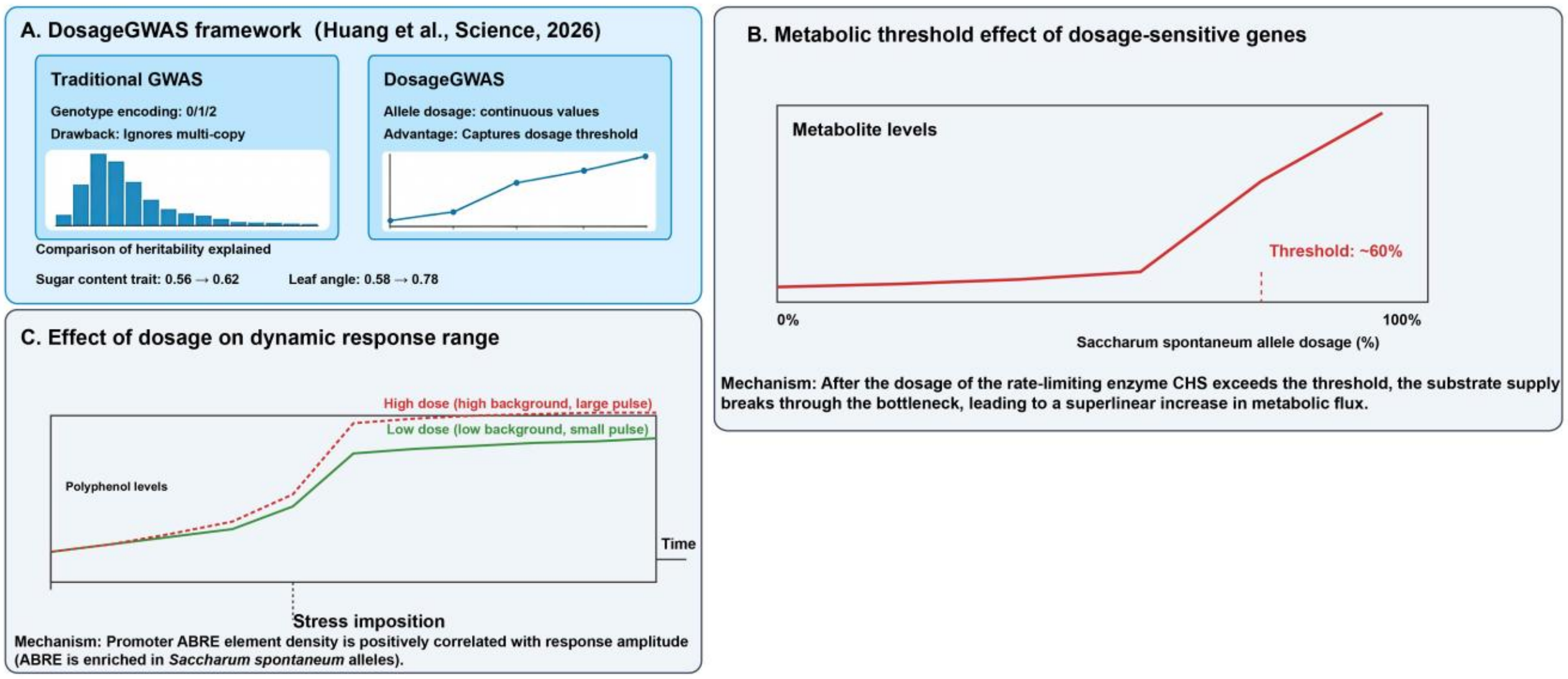

Supplementary Figure S3.

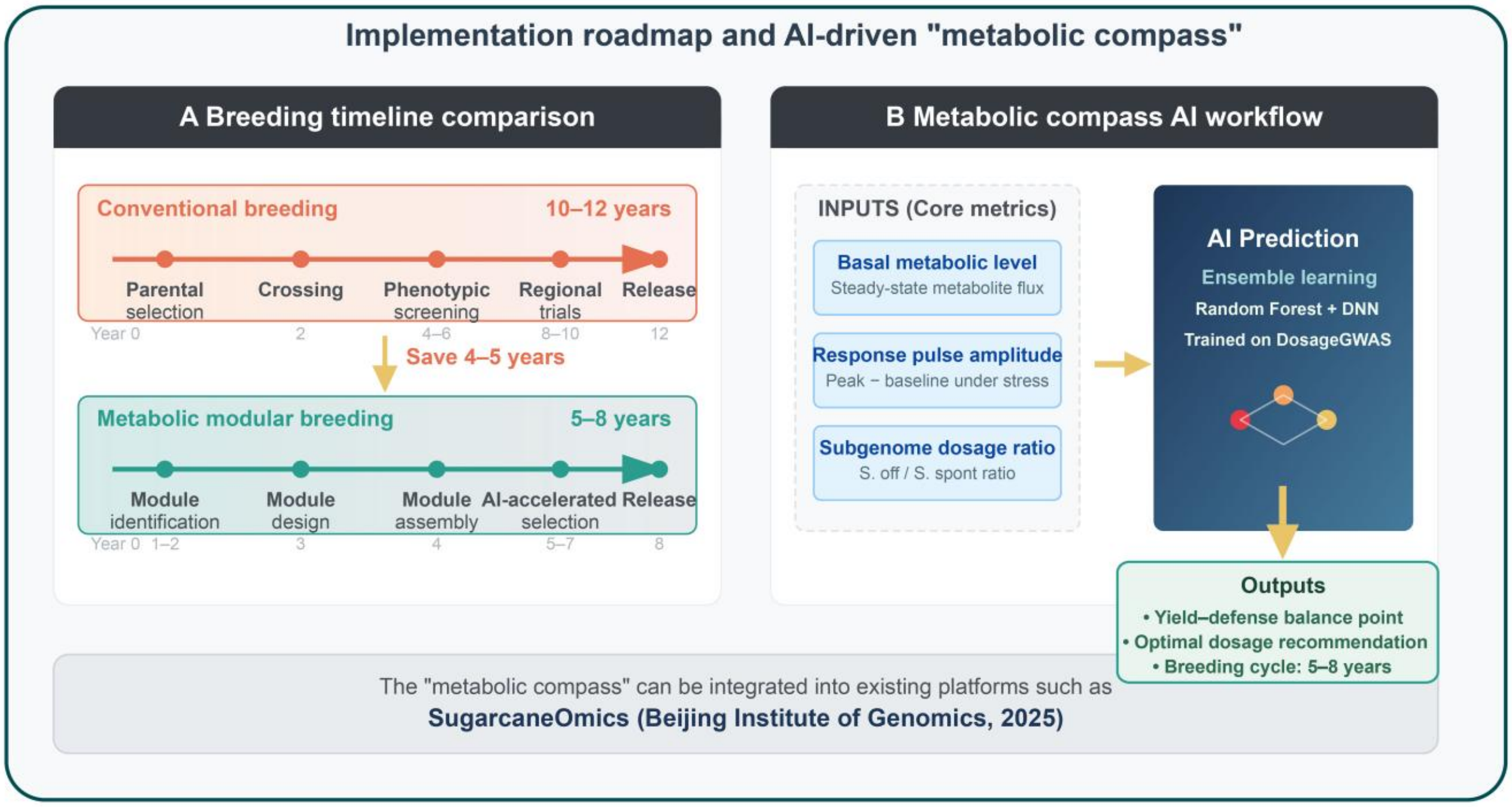

Supplement: Supplementary file 1 [file mmc1.pdf]
