# Supplementary material for: Subspecies-specific metabolic gene clusters shape polyphenol dynamics for modular metabolic breeding in sugarcane
Source: Plant Commun. 2026 Aug 6;7(9):102042. doi: 10.1016/j.xplc.2026.102042 (PMC13589566; doi:10.1016/j.xplc.2026.102042)

Plant Communications
Commentary

CellPress
Partner Journal

Subspecies-specific metabolic gene clusters shape polyphenol dynamics for modular metabolic breeding in sugarcane

Modern sugarcane cultivars are highly polyploid interspecific hybrids derived predominantly from *Saccharum officinarum* and *Saccharum spontaneum*. These divergent subgenomic contributions support high sugar yields and environmental resilience; however, they also complicate efforts to combine productivity with durable stress tolerance (Wang et al., 2023; Zhang et al., 2025). The multiscale graph pan-genome and dosage-aware genome-wide association study (DosageGWAS) framework recently developed for sugarcane now permits the analysis of subgenome-resolved variants and the quantitative estimation of allele dosage (Huang et al., 2026). This framework builds on rice pan-genome studies that revealed extensive sequence, presence/absence, and structural variation across cultivated and wild germplasm and established a super pan-genomic landscape (Zhao et al., 2018; Shang et al., 2022), while extending graph-based representation to mixed-ploidy sugarcane. Together, these resources create an opportunity to examine whether polyphenol biosynthetic genes and their regulators form subspecies-biased metabolic modules that influence the growth–defense balance.

Polyphenols participate in redox regulation, stress adaptation, and developmental signaling. In sugarcane, MYB- and WRKY-family regulators have been characterized, but the physical organization, ancestry, and dosage dependence of the corresponding pathway genes remain incompletely resolved (Li et al., 2020; Yuan et al., 2021). We therefore propose a testable framework in which candidate subspecies-specific metabolic gene clusters (MGCs) coordinate polyphenol dynamics through genomic organization, transcriptional regulation, biochemical feedback, and allele-dosage effects. We also discuss how these layers could inform modular metabolic breeding. Throughout, we distinguish established observations from hypotheses that require direct validation in sugarcane. Supplemental Table S1 defines the principal terms used in this framework.

GENOMIC ARCHITECTURE AND TRANSCRIPTIONAL REGULATORY NETWORKS OF SUBSPECIES-SPECIFIC MGCs

The sugarcane graph pan-genome projects nine genomes from four *Saccharum* species onto 10 ancestral chromosome communities, enabling alleles and structural variants to be assigned to their likely ancestral backgrounds (Huang et al., 2026). This coordinate system enables testing whether flavonoid pathway genes are physically clustered, co-inherited, or coordinately regulated in a subspecies-dependent manner. Figures 1A and 1C present two working modules: an *S. officinarum*-biased module associated with the FLS/flavonol branch and an *S. spontaneum*-biased module associated with the ANS/anthocyanin branch. These assignments represent working hypotheses rather than established MGC boundaries.

Validation requires more than colocalization. Candidate modules should be evaluated using haplotype-resolved structural variation, linkage and recombination patterns, chromatin accessibility, transcription-factor occupancy, co-expression, and metabolite quantitative-trait loci. Their activity should also be tested across tissues, developmental stages, and stress conditions. ABA, jasmonic acid, and salicylic acid signaling may converge on MYB, basic helix-loop-helix, NAC, and WRKY regulators, thereby altering pathway flux and metabolite accumulation. Sugarcane MYB and WRKY families provide plausible regulatory candidates (Li et al., 2020; Yuan et al., 2021); however, direct connections between specific hormone-responsive factors, ancestral haplotypes, and metabolic outputs remain to be demonstrated. A rigorous MGC designation should therefore require evidence of physical proximity or conserved linkage, as well as coordinated regulation and a shared metabolic function.

PUTATIVE POLYPHENOL–HORMONE–ION REGULATORY MECHANISMS

In addition to serving as pathway end products, polyphenols may modulate developmental and stress signaling. In *Arabidopsis*, flavonols stabilize PIN auxin-efflux complexes and influence polar auxin transport, providing a mechanistic precedent for feedback from specialized metabolism to growth regulation (Teale et al., 2020). Stress-induced ABA signaling may likewise increase flavonol production through SnRK2-, ABF/AREB-, and MYB-associated transcriptional programs, and accumulated flavonols can in turn alter cellular redox status. However, a direct ABA–flavonol feedback loop has not been established in sugarcane.

Guard-cell ion transport is an additional plausible interface between polyphenol accumulation and stress responses (Kashtoh and Baek, 2021). Ca^{2+}–calmodulin signaling is an established component of plant immune activation and may provide a second route linking metabolic state to stomatal or immune responses (Sun et al., 2022), but direct regulation by sugarcane polyphenols remains unproven. Supplemental Figure 1 therefore presents these interactions as testable mechanisms inferred largely from model plants. Priority experiments include ligand–protein interaction assays, channel electrophysiology, phosphoproteomics,

 © 2026 The Author(s). Published by Elsevier Inc. on behalf of CAS Center for Excellence in Molecular Plant Sciences, Chinese Academy of Sciences, and Chinese Society for Plant Biology.
This is an open access article under the CC BY license (http://creativecommons.org/licenses/by/4.0/).

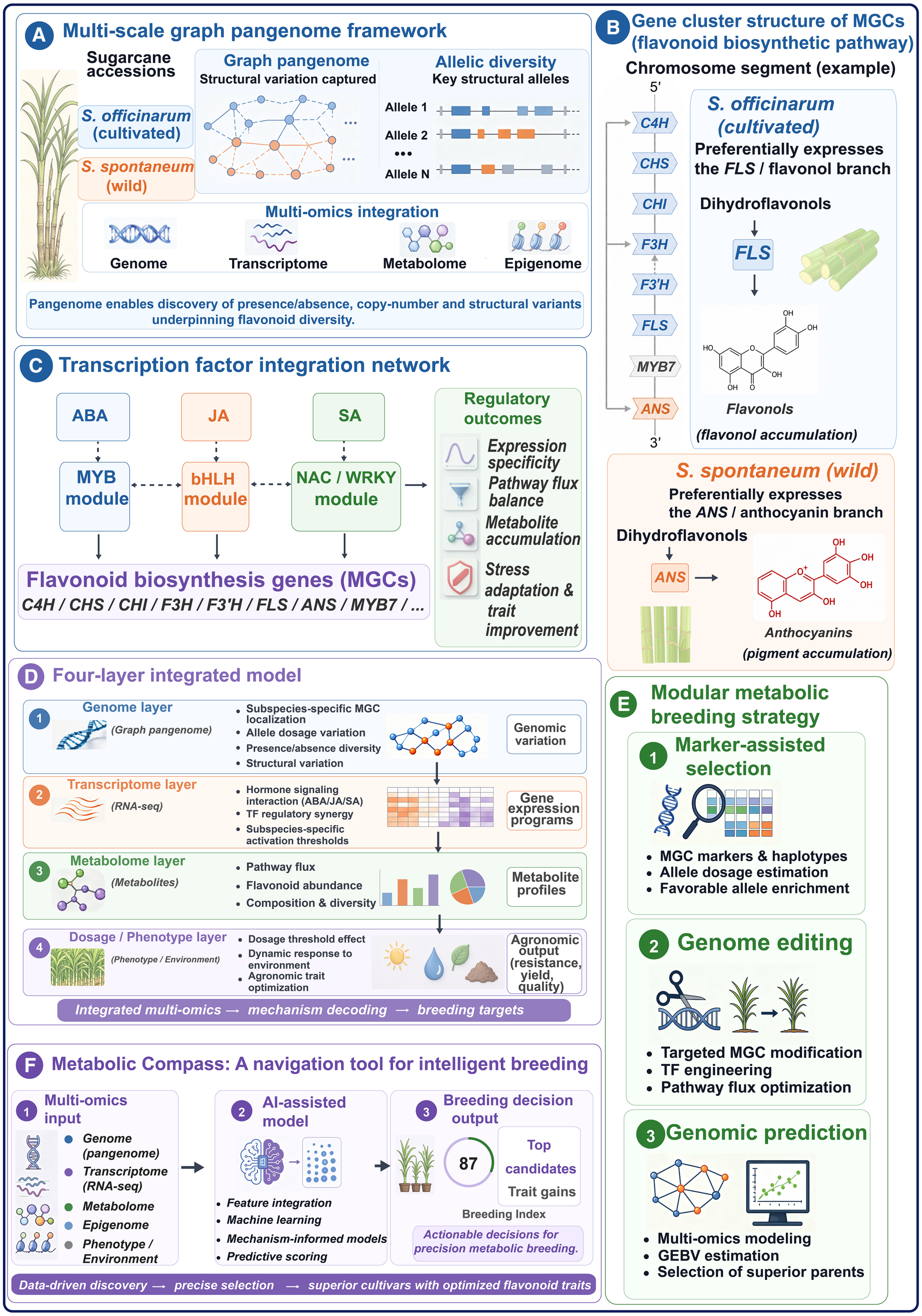

Figure 1. An integrated framework for testing subspecies-specific flavonoid metabolic gene clusters (MGCs) and guiding modular metabolic breeding in sugarcane.

(A) A multiscale graph pan-genome mapping alleles and structural variants to ancestral *Saccharum* backgrounds and integrating genomic variation with transcriptomic, metabolomic, and epigenomic information.

(legend continued on next page)

targeted gene perturbation, and metabolite add-back experiments. These experiments will help distinguish direct biochemical regulation from indirect effects arising from altered redox state, hormone abundance, or transcriptional reprogramming.

QUANTITATIVE MODELING OF POLYPLOID DOSAGE EFFECTS

In a highly polyploid genome, the presence or absence of an allele is less informative than its copy number, ancestry, and regulatory context. By estimating continuous allele dosage, DosageGWAS increased the proportion of heritability explained for representative sugarcane traits, demonstrating the analytical power of dosage-aware models (Huang et al., 2026). However, gene dosage may not translate linearly into transcript or metabolite abundance. Polyploid systems can display proportional expression, dosage compensation, homoeolog bias, and pathway-level constraints (Bekaert et al., 2011; He et al., 2022).

For candidate MGCs, the relationships between dosage and expression, metabolite abundance, and phenotype should therefore be modeled using nonlinear or piecewise functions rather than by assuming a universal threshold. Models should incorporate dosage uncertainty, ancestral origin, population structure, environment, dosage-by-environment interactions, and epistasis among homoeologs. Basal polyphenol abundance and the amplitude of stress-induced responses may serve as complementary quantitative traits: high constitutive abundance could impose a metabolic cost, whereas a rapid inducible pulse may preserve defense with lower basal expenditure. The proposed 60% *S. spontaneum* dosage threshold and 40:60 ancestral ratio shown in Supplemental Figure 2 are illustrative, hypothesis-generating values rather than validated optima. Locus-specific thresholds must be estimated empirically and replicated across genetic backgrounds and environments.

AN INTEGRATED MODEL AND MODULAR METABOLIC BREEDING STRATEGY

The proposed framework links four analytical layers (Figure 1D). The genome layer defines candidate MGC locations, ancestry, haplotype structure, and dosage. The transcriptional layer measures hormone-responsive regulatory wiring and activation thresholds. The metabolic layer quantifies pathway flux, metabolite composition, basal abundance, and inducible responses. The phenotype layer evaluates yield, sugar accumulation, quality, and stress performance. Integrating these layers can transform a descriptive pathway model into a set of testable, potentially selectable modules.

A modular metabolic breeding workflow would proceed in three stages (Figure 1E). First, graph pan-genome, transcriptomic, and metabolomic data would be integrated to identify candidate modules and prioritize alleles with reproducible effects. Second, genome editing or *cis*-regulatory engineering would be used to modify specific nodes while preserving the remainder of the pathway (Li et al., 2024). Regulatory-element editing has been used to decouple yield components in rice, and targeted editing of metabolic pathways has altered gossypol composition in cotton, demonstrating the feasibility of trait-specific metabolic redesign (Song et al., 2022; Lin et al., 2023). In sugarcane, editing strategies must account for allele dosage, homoeolog redundancy, off-target effects, and the possibility that linked genes within an MGC may be inherited as a unit. Recombination frequency and epistatic interactions among ancestral alleles should therefore also be considered in module design.

Third, dosage-aware genomic prediction could evaluate combinations that are impractical to test exhaustively in the field. We term the resulting decision framework a "metabolic compass" (Figure 1F and Supplemental Figure 3): a model that combines basal metabolic levels, stress-induced response amplitude, allele dosage, environment, and agronomic performance. It should not prescribe a universal ancestral ratio. Instead, it should generate uncertainty-calibrated predictions for specific populations and target environments. CRISPR and other genome-modification tools can then be used to test high-priority predictions rather than directly implementing unvalidated dosage targets. Prospective validation should compare edited or selected lines across environments and measure both agronomic benefits and potential metabolic costs. An illustrative implementation roadmap and AI-assisted prediction workflow are presented in Supplemental Figure 3.

CONCLUDING REMARKS

The sugarcane graph pan-genome and DosageGWAS framework enable polyphenol regulation to be investigated at subgenome and copy-number resolution. We propose that candidate subspecies-specific MGCs may link ancestral genome structure to transcriptional regulation, metabolic feedback, and quantitative dosage effects. This model offers a coherent route from association to mechanism, but its central elements—including MGC boundaries, branch-specific ancestry, hormone–ion feedback, and dosage thresholds—remain hypothetical and require experimental validation.

Modular metabolic breeding should therefore follow an iterative design–test–learn strategy. Its success will depend on accurate dosage estimation, causal validation, explicit modeling of

(B) Candidate pathway modules comprising an *S. officinarum*-biased FLS/flavonol branch and an *S. spontaneum*-biased ANS/anthocyanin branch. These branch assignments are working hypotheses rather than validated MGC boundaries.
(C) ABA, jasmonic acid, and salicylic acid signaling may converge on MYB, basic helix-loop-helix, NAC, and WRKY regulators to influence pathway activity, metabolite accumulation, and stress responses.
(D) A four-layer model integrating genomic architecture and dosage, transcriptional regulation, metabolic output, and agronomic phenotype.
(E) Proposed breeding workflow combining module identification, targeted editing or *cis*-regulatory engineering, and dosage-aware genomic prediction. Recombination, linkage, and interactions among ancestral alleles must be considered in module design.
(F) The "metabolic compass" integrates basal metabolite abundance, stress-induced response amplitude, allele dosage, environment, and agronomic performance to inform population- and environment-specific predictions. This conceptual decision framework does not prescribe a universal ancestral dosage ratio.

recombination and allele interactions, and field testing across environments. If these requirements are met, the framework could help identify polyphenol configurations that preserve inducible defense while limiting unnecessary constitutive metabolic costs, thereby supporting precision improvement in complex polyploid crops.

ACKNOWLEDGMENTS

We thank the editor and three reviewers for their constructive evaluation of this commentary. We also thank Muqing Zhang and colleagues for discussions that informed the polyphenol–hormone–ion framework, the Sugarcane Breeding Innovation Team at Guangxi University for insights into polyploid genetics and dosage effects, and Jishuang Chen for comments on MGCs.

Funding: this work was supported by the National Natural Science Foundation of China (grant no. U24A20387 to J.Z.), the Guangxi Science and Technology Major Program (grant no. AB24153006 and AA24206023 to J.Z.), and the National Key Research and Development Program (grant no. 2024YFF1000800).

AUTHOR CONTRIBUTIONS

J.Z., conceptualization, supervision, funding acquisition, and writing – review and editing; D.W., investigation, data curation, formal analysis, and writing – original draft; D.F., methodology, investigation, and writing – review and editing. All authors have read and approved the manuscript.

SUPPLEMENTAL INFORMATION

Supplemental information is available at *Plant Communications Online*.

Received: April 30, 2026
Revised: June 5, 2026
Accepted: August 5, 2026
Published: August 6, 2026

Dongdong Wang[1], Ding Fan[1] and Jisen Zhang[1,]*

[1]Guangxi Sugarcane Bio-Breeding Laboratory, State Key Lab for Conservation and Utilization of Subtropical Agro-Biological Resources, College of Agriculture, Guangxi University, Nanning, China
***Correspondence: Jisen Zhang (zjisen@gxu.edu.cn)**
https://doi.org/10.1016/j.xplc.2026.102042

REFERENCES

Bekaert, M., Edger, P.P., Pires, J.C., and Conant, G.C. (2011). Two-phase resolution of polyploidy in the Arabidopsis metabolic network gives rise to relative and absolute dosage constraints. Plant Cell **23**:1719–1728. https://doi.org/10.1105/tpc.110.081281.

He, F., Wang, W., Rutter, W.B., Jordan, K.W., Ren, J., Taagen, E., DeWitt, N., Sehgal, D., Sukumaran, S., Dreisigacker, S., et al. (2022). Genomic variants affecting homoeologous gene expression dosage contribute to agronomic trait variation in allopolyploid wheat. Nat. Commun. **13**:826. https://doi.org/10.1038/s41467-022-28453-y.

Huang, Y., Zhang, Y., Zhang, Q., Zhuang, G., Li, C., Wang, B., Gao, R., Xu, Y., Qi, Y., Hua, X., et al. (2026). Multiscale pangenome graphs empower the genomic dissection of mixed-ploidy sugarcane species. Science **391**:eadx1616. https://doi.org/10.1126/science.adx1616.

Kashtoh, H., and Baek, K.-H. (2021). Structural and functional insights into the role of guard cell ion channels in abiotic stress-induced stomatal closure. Plants **10**:2774. https://doi.org/10.3390/plants10122774.

Li, B., Sun, C., Li, J., and Gao, C. (2024). Targeted genome-modification tools and their advanced applications in crop breeding. Nat. Rev. Genet. **25**:603–622. https://doi.org/10.1038/s41576-024-00720-2.

Li, Z., Hua, X., Zhong, W., Yuan, Y., Wang, Y., Wang, Z., Ming, R., and Zhang, J. (2020). Genome-wide identification and expression profile analysis of WRKY family genes in the autopolyploid Saccharum spontaneum. Plant Cell Physiol. **61**:616–630. https://doi.org/10.1093/pcp/pcz227.

Lin, J.-L., Fang, X., Li, J.-X., Chen, Z.-W., Wu, W.-K., Guo, X.-X., Liu, N.J., Huang, J.F., Chen, F.Y., Wang, L.J., et al. (2023). Dirigent gene editing of gossypol enantiomers for toxicity-depleted cotton seeds. Nat. Plants **9**:605–615. https://doi.org/10.1038/s41477-023-01376-2.

Shang, L., Li, X., He, H., Yuan, Q., Song, Y., Wei, Z., Lin, H., Hu, M., Zhao, F., Zhang, C., et al. (2022). A super pan-genomic landscape of rice. Cell Res. **32**:878–896. https://doi.org/10.1038/s41422-022-00685-z.

Song, X., Meng, X., Guo, H., Cheng, Q., Jing, Y., Chen, M., Liu, G., Wang, B., Wang, Y., Li, J., et al. (2022). Targeting a gene regulatory element enhances rice grain yield by decoupling panicle number and size. Nat. Biotechnol. **40**:1403–1411. https://doi.org/10.1038/s41587-022-01281-7.

Sun, L., Qin, J., Wu, X., Zhang, J., and Zhang, J. (2022). TOUCH 3 and CALMODULIN 1/4/6 cooperate with calcium-dependent protein kinases to trigger calcium-dependent activation of CAM-BINDING PROTEIN 60-LIKE G and regulate fungal resistance in plants. Plant Cell **34**:4088–4104. https://doi.org/10.1093/plcell/koac209.

Teale, W.D., Pasternak, T., Dal Bosco, C., Dovzhenko, A., Kratzat, K., Bildl, W., Schwörer, M., Falk, T., Ruperti, B., Schaefer, J.V., et al. (2021). Flavonol-mediated stabilization of PIN efflux complexes regulates polar auxin transport. EMBO J. **40**:e104416. https://doi.org/10.15252/embj.2020104416.

Wang, T., Wang, B., Hua, X., Tang, H., Zhang, Z., Gao, R., Qi, Y., Zhang, Q., Wang, G., Yu, Z., et al. (2023). A complete gap-free diploid genome in Saccharum complex and the genomic footprints of evolution in the highly polyploid Saccharum genus. Nat. Plants **9**:554–571. https://doi.org/10.1038/s41477-023-01378-0.

Yuan, Y., Yang, X., Feng, M., Ding, H., Khan, M.T., Zhang, J., and Zhang, M. (2021). Genome-wide analysis of R2R3-MYB transcription factors family in the autopolyploid Saccharum spontaneum: an exploration of dominance expression and stress response. BMC Genom. **22**:622. https://doi.org/10.1186/s12864-021-07689-w.

Zhang, J., Qi, Y., Hua, X., Wang, Y., Wang, B., Qi, Y., Huang, Y., Yu, Z., Gao, R., Zhang, Y., et al. (2025). The highly allo-autopolyploid modern sugarcane genome and very recent allopolyploidization in Saccharum. Nat. Genet. **57**:242–253. https://doi.org/10.1038/s41588-024-02033-w.

Zhao, Q., Feng, Q., Lu, H., Li, Y., Wang, A., Tian, Q., Zhan, Q., Lu, Y., Zhang, L., Huang, T., et al. (2018). Pan-genome analysis highlights the extent of genomic variation in cultivated and wild rice. Nat. Genet. **50**:278–284. https://doi.org/10.1038/s41588-018-0041-z.

Plant Communications, Volume *7*

Supplemental information

Subspecies-specific metabolic gene clusters shape polyphenol dynamics for modular metabolic breeding in sugarcane

Dongdong Wang, Ding Fan, and Jisen Zhang

Supplemental Information

Subspecies-specific metabolic gene clusters orchestrate polyphenol dynamics for modular metabolic breeding in sugarcane

Dongdong Wang[1], Ding Fan[1], Jisen Zhang[1]*

[1] Guangxi Sugarcane Bio-Breeding Laboratory, State Key Lab for Conservation and Utilization of Subtropical Agro-Biological Resources, College of Agriculture, Guangxi University, Nanning, China

*Correspondence: Jisen Zhang (zjisen@gxu.edu.cn)

Supplemental Note S1. Evidence and validation requirements for putative polyphenol–hormone–ion interactions

Figure S1 separates evidence established in model plants from mechanisms proposed for sugarcane. Flavonol-dependent stabilization of PIN auxin-efflux complexes provides direct evidence that a specialized metabolite can alter hormone transport (Teale et al., 2020). This observation supports, but does not demonstrate, a comparable flavonol–auxin module in sugarcane. Validation should combine sugarcane PIN homolog identification, subcellular localization, phosphosite analysis, ligand-binding assays and auxin-transport measurements after genetic or chemical manipulation of flavonol abundance.

ABA-associated flavonol accumulation is presented as a regulatory hypothesis. A defensible test should distinguish transcriptional induction of biosynthesis from direct modulation of ABA receptors or signaling proteins. Time-resolved ABA, ROS and flavonol measurements, together with perturbation of PYR/PYL–PP2C–SnRK2 and MYB/ABF components, would establish directionality. Rescue experiments should determine whether altered flavonol abundance restores the relevant phenotype independently of broader stress-induced transcriptional changes.

Guard-cell ion transport is a plausible interface because K^+ and other channels regulate

stomatal responses (Kashtoh and Baek, 2021). Ca^{2+}–calmodulin signaling is required for important plant immune outputs and provides an additional testable route linking metabolic state with stress signaling (Sun et al., 2022), but direct control by sugarcane polyphenols remains unproven. Electrophysiology, purified-protein binding, dose–response analysis and genetic epistasis are required before these interactions can be described as causal sugarcane mechanisms.

Supplemental Note S2. Assumptions for dosage-aware analysis of candidate MGCs

DosageGWAS provides a quantitative basis for associating allele copy number with complex traits in mixed-ploidy sugarcane (Huang et al., 2026). For candidate MGCs, however, dosage should be propagated as an estimate with uncertainty rather than forced into an error-free integer. Models should jointly consider ancestry, local haplotype structure, population structure, environmental covariates and relatedness.

The expected relationship among dosage, expression and metabolite abundance may be linear, saturating, threshold-like or compensated. Evidence from polyploid metabolic networks and wheat homoeolog expression shows that dosage sensitivity is locus- and pathway-dependent (Bekaert et al., 2011; He et al., 2022). Accordingly, linear, spline and piecewise models should be compared, with out-of-sample validation and uncertainty intervals. Dosage-by-environment and dosage-by-dosage interactions should be tested when sample size permits.

Figure S2 uses basal abundance and stress-induced pulse amplitude as conceptual phenotypes. The displayed 60% *S. spontaneum* value is an illustrative reference point, not a threshold established by Huang et al. (2026) or by direct sugarcane CHS experiments. Likewise, a 40:60 *S. officinarum*:*S. spontaneum* ratio must not be interpreted as a universal breeding optimum. Any threshold or optimum must be estimated at a defined locus, in a defined population and environment, and replicated independently.

Supplemental Note S3. Implementation of modular metabolic breeding and the metabolic compass

The proposed workflow has three iterative stages. Module identification integrates graph-pangenome haplotypes, structural variants, expression, chromatin features, metabolite profiles and phenotypes, building on chromosome-resolved Saccharum genomic resources (Wang et al., 2023; Huang et al., 2026). Candidate modules should be prioritized only when ancestry and dosage effects are reproducible and when physical linkage or coordinated regulation supports an MGC interpretation.

Module design uses genome editing, cis-regulatory engineering or allele selection to perturb a defined node (Li et al., 2024). Examples in rice and cotton show that regulatory and metabolic traits can be redesigned without altering all pathway functions (Song et al., 2022; Lin et al., 2023). Sugarcane applications must additionally address high ploidy, homoeolog redundancy, mosaic editing and linkage among neighboring genes. Each design therefore requires molecular characterization, dosage verification and multi-environment field validation.

Module assembly combines dosage-aware prediction with multi-environment phenotyping. The metabolic compass should output predicted benefit, metabolic cost and uncertainty rather than a single fixed ratio. Training and validation datasets must be separated, and model performance should be evaluated across genetic backgrounds and target environments. The timelines shown in Figure S3 are illustrative planning scenarios rather than demonstrated reductions in breeding duration.

Supplemental Table S1. Glossary of key terms for modular metabolic breeding

Term	Definition
Metabolic gene cluster (MGC)	A physically linked or conserved set of genes that contributes to a shared metabolic function. In this Commentary, the proposed sugarcane MGCs remain candidate modules pending structural and functional validation.

Term	Definition
DosageGWAS	A dosage-aware association framework that estimates allele dosage in polyploid genomes and tests its relationship with quantitative traits.
Basal metabolic level	Metabolic abundance under a defined non-stressed condition. Its interpretation requires standardized tissue, developmental stage and environment.
Response pulse amplitude	The stress-induced change relative to the basal level, quantified over a defined time course.
Metabolic compass	A conceptual prediction framework integrating metabolic state, allele dosage, environment and agronomic performance with calibrated uncertainty.
Yield–defense balance point	A population-, locus- and environment-specific compromise between productivity and defense. It is not assumed to correspond to a universal ancestral dosage ratio.

Supplementary Figure legends

Supplementary Figure S1. Putative polyphenol-mediated regulation of hormone and ion signaling.

A. Model-plant evidence indicates that flavonols can stabilize PIN auxin-efflux complexes and alter polar auxin transport. B. ABA signaling may induce flavonol biosynthesis, whereas flavonol-dependent redox changes may feed back on stress responses. C. Guard-cell K^+ channels and Ca^{2+}–calmodulin signaling are proposed as additional interfaces. The pathways in B and C remain to be validated directly in sugarcane.

Supplementary Figure S2. Conceptual modeling of polyploid dosage effects.

A. DosageGWAS estimates continuous allele dosage and tests dosage–trait associations. B. A nonlinear dosage–metabolite relationship is shown as an illustrative model; the plotted 60% *S. spontaneum* value is not a validated CHS threshold. C. Basal metabolic level and stress-induced pulse amplitude are proposed as complementary quantitative traits. The direction and magnitude of these relationships must be estimated empirically for each locus and environment.

Supplementary Figure S3. Implementation roadmap and AI-assisted metabolic compass for modular metabolic breeding in sugarcane.

A. Comparison of a conventional breeding workflow with a proposed modular workflow comprising module identification, module design and module assembly. The displayed durations are illustrative planning scenarios, not demonstrated reductions in breeding time. B. The metabolic compass integrates basal metabolic level, response pulse amplitude, allele dosage, environment and field performance in an uncertainty-calibrated prediction model.

Supplemental References

Bekaert, M., Edger, P.P., Pires, J.C., and Conant, G.C. (2011). Two-phase resolution of polyploidy in the *Arabidopsis* metabolic network gives rise to relative and absolute dosage constraints. Plant Cell 23, 1719–1728. https://doi.org/10.1105/tpc.110.081281.

He, F., Wang, W., Rutter, W.B., Jordan, K.W., Ren, J., Taagen, E., et al. (2022). Genomic variants affecting homoeologous gene expression dosage contribute to agronomic trait variation in allopolyploid wheat. Nature Communications 13, 826. https://doi.org/10.1038/s41467-022-28453-y.

Huang, Y., Zhang, Y., Zhang, Q., Zhuang, G., Li, C., Wang, B., et al. (2026). Multiscale pangenome graphs empower the genomic dissection of mixed-ploidy sugarcane species. Science 391, eadx1616. https://doi.org/10.1126/science.adx1616.

Kashtoh, H., and Baek, K.-H. (2021). Structural and functional insights into the role of guard cell ion channels in abiotic stress-induced stomatal closure. Plants 10, 2774. https://doi.org/10.3390/plants10122774.

Li, B., Sun, C., Li, J., and Gao, C. (2024). Targeted genome-modification tools and their advanced applications in crop breeding. Nature Reviews Genetics 25, 603–622. https://doi.org/10.1038/s41576-024-00720-2.

Li, Z., Hua, X., Zhong, W., Yuan, Y., Wang, Y., Wang, Z., et al. (2020). Genome-wide identification and expression profile analysis of WRKY family genes in the autopolyploid *Saccharum spontaneum*. Plant and Cell Physiology 61, 616–630. https://doi.org/10.1093/pcp/pcz227.

Lin, J.-L., Fang, X., Li, J.-X., Chen, Z.-W., Wu, W.-K., Guo, X.-X., et al. (2023). Dirigent gene editing of gossypol enantiomers for toxicity-depleted cotton seeds. Nature Plants 9, 605–615. https://doi.org/10.1038/s41477-023-01376-2.

Shang, L., Li, X., He, H., Yuan, Q., Song, Y., Wei, Z., et al. (2022). A super pan-genomic landscape of rice. Cell Research 32, 878–896. https://doi.org/10.1038/s41422-022-00685-z.

Song, X., Meng, X., Guo, H., Cheng, Q., Jing, Y., Chen, M., et al. (2022). Targeting a gene regulatory element enhances rice grain yield by decoupling panicle number and size. Nature Biotechnology 40, 1403–1411. https://doi.org/10.1038/s41587-022-01281-7.

Sun, L., Qin, J., Wu, X., Zhang, J., and Zhang, J. (2022). TOUCH 3 and CALMODULIN 1/4/6 cooperate with calcium-dependent protein kinases to trigger calcium-dependent activation of CAM-BINDING PROTEIN 60-LIKE G and regulate fungal resistance in plants. Plant Cell 34, 4088–4104. https://doi.org/10.1093/plcell/koac209.

Teale, W.D., Pasternak, T., Dal Bosco, C., Dovzhenko, A., Kratzat, K., Bildl, W., et al. (2020). Flavonol-mediated stabilization of PIN efflux complexes regulates polar auxin transport. EMBO Journal 40, e104416. https://doi.org/10.15252/embj.2020104416.

Wang, T., Wang, B., Hua, X., Tang, H., Zhang, Z., Gao, R., et al. (2023). A complete gap-free diploid genome in *Saccharum* complex and the genomic footprints of evolution in the highly polyploid *Saccharum* genus. Nature Plants 9, 554–571. https://doi.org/10.1038/s41477-023-01378-

0.

Yuan, Y., Yang, X., Feng, M., Ding, H., Khan, M.T., Zhang, J., et al. (2021). Genome-wide analysis of R2R3-MYB transcription factors family in the autopolyploid *Saccharum spontaneum*: an exploration of dominance expression and stress response. BMC Genomics 22, 622. https://doi.org/10.1186/s12864-021-07689-w.

Zhang, J., Qi, Y., Hua, X., Wang, Y., Wang, B., Qi, Y., et al. (2025). The highly allo-autopolyploid modern sugarcane genome and very recent allopolyploidization in *Saccharum*. Nature Genetics 57, 242–253. https://doi.org/10.1038/s41588-024-02033-w.

Zhao, Q., Feng, Q., Lu, H., Li, Y., Wang, A., Tian, Q., et al. (2018). Pan-genome analysis highlights the extent of genomic variation in cultivated and wild rice. Nature Genetics 50, 278–284. https://doi.org/10.1038/s41588-018-0041-z.

Supplementary Figure S1.

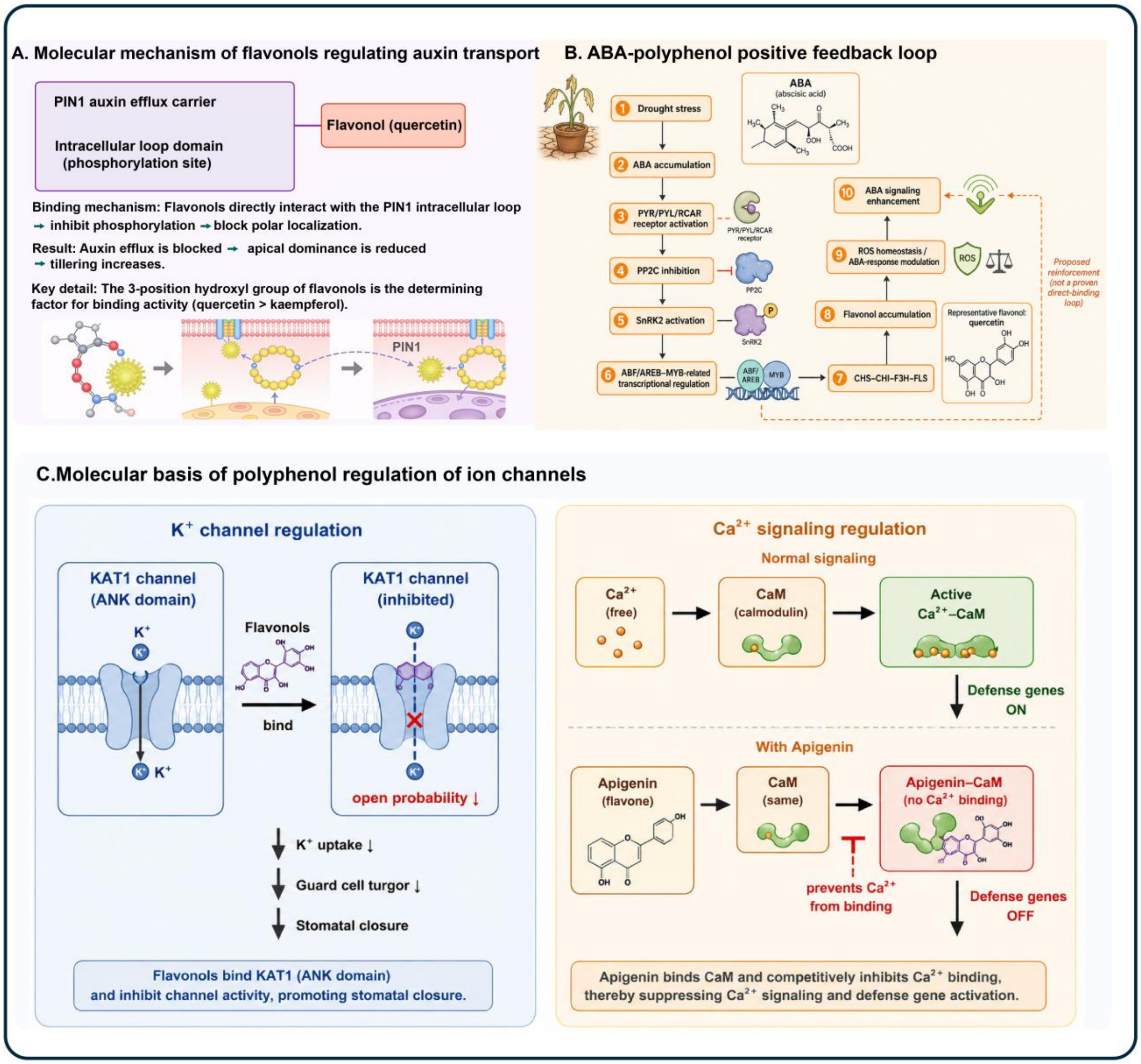

Supplementary Figure S2.

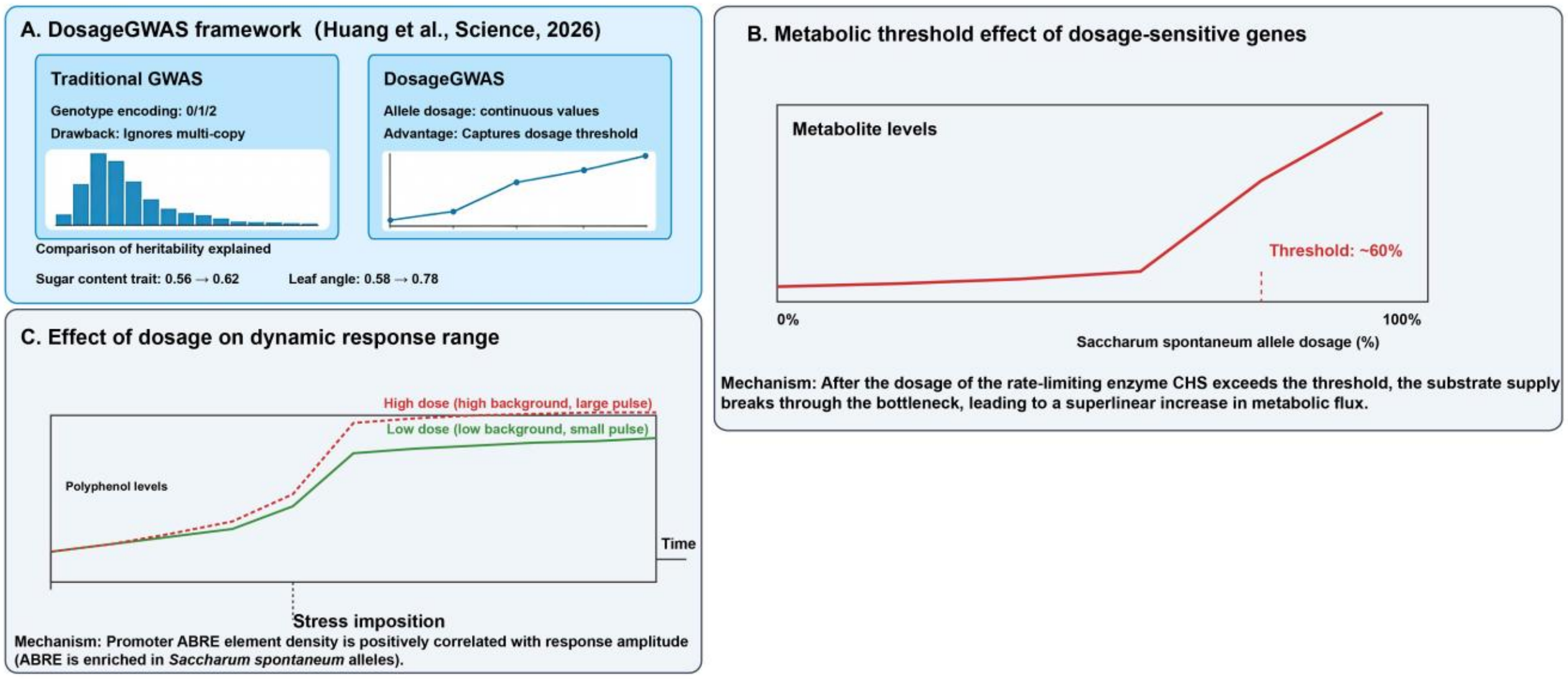

Supplementary Figure S3.

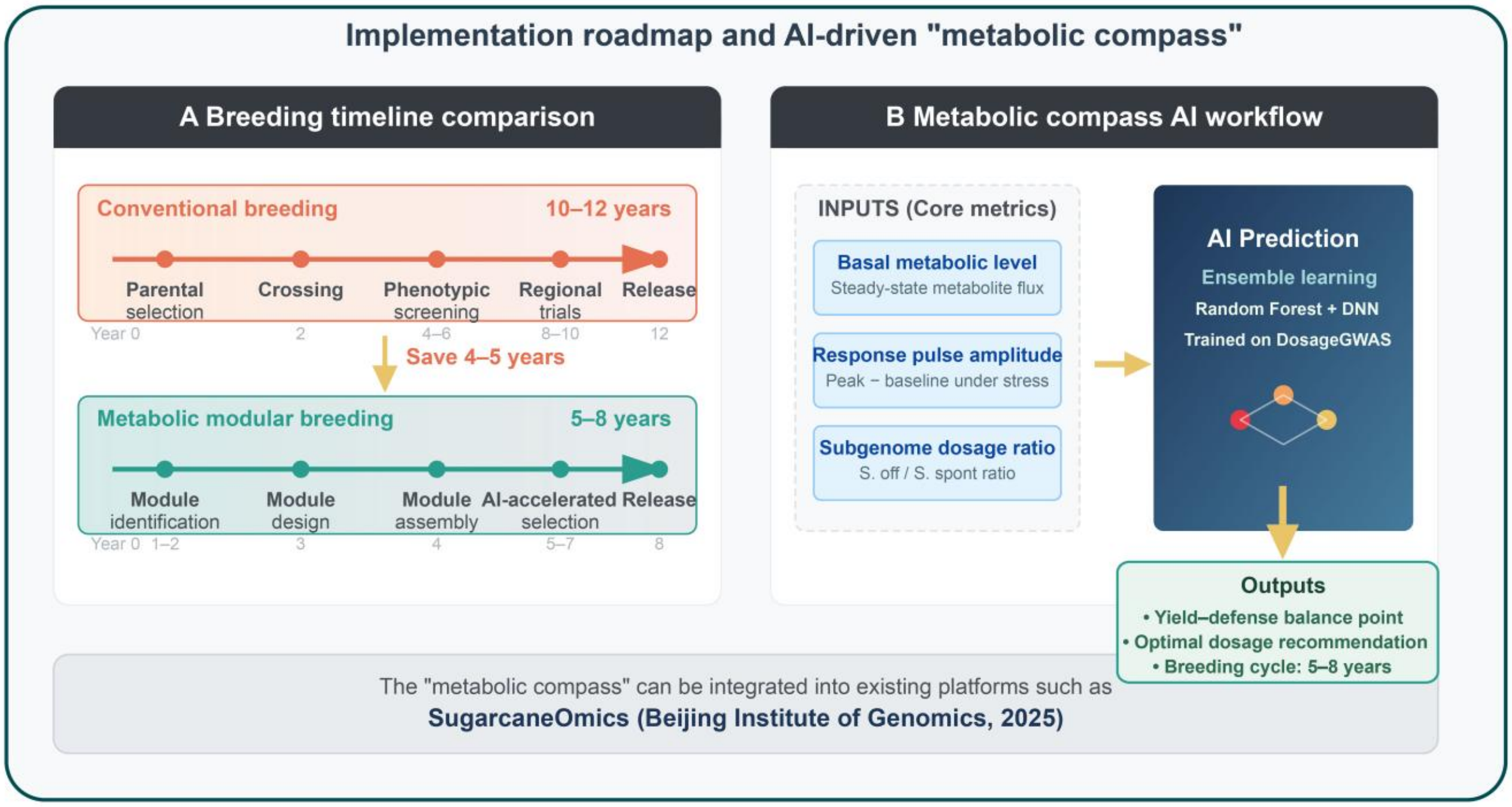

Supplement: Supplementary file 2 [file mmc2.pdf]
